# Hybrid recommender system model for digital library from multiple online publishers

**DOI:** 10.12688/f1000research.133013.2

**Published:** 2024-04-04

**Authors:** Pijitra Jomsri, Dulyawit Prangchumpol, Kittiya Poonsilp, Thammarat Panityakul

**Affiliations:** 1 Suan Sunandha Rajabhat University, Dusit, Bangkok, 10300, Thailand; 2 Prince of Songkla University, Hat Yai, Songkhla, 90110, Thailand

**Keywords:** Recommender systems, digital library, multiple database, user profile, hybrid recommender systems, collaborative filtering, content-based filtering

## Abstract

**Background:**

The demand for online education promotion platforms has increased. In addition, the digital library system is one of the many systems that support teaching and learning. However, most digital library systems store books in the form of libraries that were developed or purchased exclusively by the library, without connecting data with different agencies in the same system.

**Methods:**

A hybrid recommender system model for digital libraries, developed from multiple online publishers, has created a prototype digital library system that connects various important knowledge sources from multiple digital libraries and online publishers to create an index and recommend e-books. The developed system utilizes an API-based linking process to connect various important sources of knowledge from multiple data sources such as e-books on education from educational institutions, e-books from government agencies, and e-books from religious organizations are stored separately. Then, a hybrid recommender system suitable for users was developed using Collaborative Filtering (CF) model together with Content-Based Filtering. This research purposed the hybrid recommender system model, which took into account the factors of book category, reading habits of users, and sources of information. The evaluation of the experiments involved soliciting feedback from system users and comparing the results with conventional recommendation methods.

**Results:**

A comparison of NDCG scores was conducted for Hybrid Score 50:50, Hybrid Score 20:80, Hybrid Score 80:20, CF-score and CB-score. The experimental result was found that the Hybrid Score 80:20 method had the highest average NDCG score.

**Conclusions:**

Using a hybrid recommender system model that combines 80% Collaborative Filtering and 20% Content-Based Filtering can improve the recommender method, leading to better referral efficiency and greater overall efficiency compared to traditional approaches.

## 1. Introduction

Reading is important for human development in terms of education, career development, quality of life, and national development. In Bangkok, Thailand, there are areas for self-learning through books, or public libraries, available free of charge. Libraries are necessary for people at all levels to use their knowledge from books to improve themselves, enhance their quality of life, create equality, and promote reading to the public. In addition, data published by the World Bank, UNESCO, and the United Nations (UN) demographic data indicate that the Covid-19 outbreak has contributed to over 17% of children worldwide facing a learning crisis. This may affect the potential of the modern population. Also, schools around the world have had to close more than usual over the past year. School closures have resulted in students having to switch to online classes, but the learning system does not cover the world, and many children do not have access to technology to study online, ^1^^,^^2^ which may result in a lack of basic literacy skills.

Currently, technology and telecommunication play an essential role in human life, coupled with rapidly advancing computer technology and communication systems. Therefore, the digital library system is another channel for collecting information or electronic books from multiple sources and disseminating them through the Internet. This allows children and general readers to access and search for books through a computer network without any restrictions on location, distance, and duration. It is an opportunity to expand the results of learning resources from physical to online, without borders, to expand opportunities and increase access to books, media, and publications, promoting reading widely and in line with modern society. However, the current online library system is not user-friendly because e-books are stored scattered in separate databases developed by each agency and this causes users to have to download multiple applications for reading e-books. In addition, most government economic development plans push for the promotion of reading and learning through modern regional library services, creating opportunities for youth groups to have access to quality services that are convenient and fast-quality services.

Based on such problems, the researcher has objective to developed a digital library model by studying techniques for combining multiple e-book database systems. Additionally, the researcher aims to use content to create hybrid recommender systems model for electronic books from multiple structures to serve users in the Bangkok area. This is to enable users to easily access them through a single system. Books were gathered from many important sources, and this system was able to enable the public to access the library system in an online format by designing a database system that linked electronic books from multiple databases together, collecting book information from many sources such as the National Library, government agencies, teaching materials, Dhamma books, novels, and short stories. Moreover, this research presents a model for recommending electronic books to users using the hybrid recommender systems model, combining Collaborative Filtering (CF), heuristic Content-based filtering, and user’s personal data. The researcher believes that hybrid recommender system model can enhance the efficiency of book recommendations in the digital library system. The collaborative filtering of this research concentrates on user reading, while Content-based filtering concentrates on titles, authors, book categories, keywords, and book details to offer suggestions to users in the area of their interest.

The structure of this paper is as follows: Section 2 provides a background and relevant literature. Section 3 outlines the methodology and framework employed in the hybrid recommender system. Section 4 shows the experimental outcomes. Lastly, Section 5 concludes the research and offers recommendations for future research.

## 2. Literature review

Recommender systems are ubiquitous on the internet. Typically, news websites feature a banner that displays recommendations such as “You may also like” or “People who liked this article also enjoyed this one.” This approach aligns with the traditional definition of recommender systems as outlined by Resnick and Varian, ^3^ that they are systems that study a user’s preferences for a given object to make suggestions that might be useful to the user. Recommender systems enable users to customize their profiles, receive tailored suggestions, and make informed decisions about products and services that align with their preferences. The five primary recommendation techniques include: collaborative filtering, content filtering, demographic filtering, knowledge-based filtering, and utility-based filtering. ^4^ A more fundamental way of categorizing recommender systems is to divide techniques into three primary groups: collaborative filtering, content-based filtering, and hybrid approaches. ^5^

• Content-based filtering utilizes the "Content" feature of an item to generate user profiles based on their preferences and selections. This technique suggests a list of items that are similar to those that a user has already viewed or appreciated. ^6^^–^^8^
• Collaborative filtering relies on the exchange of opinions and feedback among users. This technique suggests a list of items that have been favored by other users with similar preferences. ^9^^–^^11^
• The Hybrid Approach is a blend of Content-based and Collaborative Filtering that leverages both user preferences and item attributes. This technique utilizes a matrix derived from filtering interactions and contextual data from Content-based filtering to provide personalized recommendations. ^12^^–^^14^

In general, most databases are stored separately for different service providers. Users must know the source and explore various topics of interest. ^15^^–^^22^ However, some researchers have concluded that a single database is not sufficient to retrieve knowledge for users. Several referral systems for digital libraries have been proposed. Many researchers apply a hybrid model to improve recommender systems. Porcel *et al.* propose a hybrid system by combines collaborative recommendations and content-based. ^23^ Tejeda-Lorente *et al.* present a quality-based recommender system that considers the quality of an item in order to assess its relevance. ^24^ Serrano-Guerrero *et al.* present a fuzzy linguistic recommender model in a university digital library. This model uses the Google Wave approach that provides a shared space for different users and resources. ^25^ Morawski *et al.* offer a hybrid recommender system for rural libraries by combining content-based and collaborative filtering. The authors suggest the concept of a fuzzy flavor vector to deal with the problem of "cold start" problems caused by the smaller size of this library and the usual sparse data sets. ^26^ Jomsri proposes a library book recommendation system based on user profiling and association rules. ^27^ Some researchers focus a patron-driven hybrid library recommender system by applying machine learning techniques to recommend weeding decision-making operations by extracting and analyzing users’ opinions and ratings. ^28^

Some researchers have tried to develop models for library services, such as Yang and Hung’s proposed recommender system for book acquisition in libraries. The authors employ a basic metric that does not consider user feedback or opinions. ^29^ Wu *et al.* have introduced a library book acquisition recommender system that employs a network ranking mechanism. ^30^ Cabrerizo *et al.* suggest an extension to the LibQUAL+ model to address users’ perceptions and evaluate the quality of library services. ^31^^–^^32^ Some researchers use linked information spaces for different scientific digital libraries in Digital Humanities. ^33^ Another researcher conducted a study with the aim of developing a recommendation system model that integrates various types of supplementary information, apart from explicit ratings assigned to items. This supplementary information includes social connections between users and data on the items being recommended. ^34^ The main aim of researching the Hybrid Recommendation model is to overcome the issue of insufficient rating data by integrating the information from Content-Based and Collaborative Filtering models. Numerous studies have been conducted in this area, including one that implemented the Bayesian Probabilistic Matrix Factorization Framework to tackle the sparsity problem by supplementing taste data with user evaluation data stored in a matrix. Another study utilized an auto-encoder to learn side information data when user preference information is inadequate. Furthermore, a study was carried out to integrate information by utilizing an automatic encoder to learn the nonlinear activity of users and items while removing stacked noise. ^35^^,^^36^ The technique for recommender in this paper applies a hybrid approach model and creates an API for connecting content from multiple e-book databases to recommend users.

## 3. Methodology and framework of hybrid recommender system

**Figure 1.**
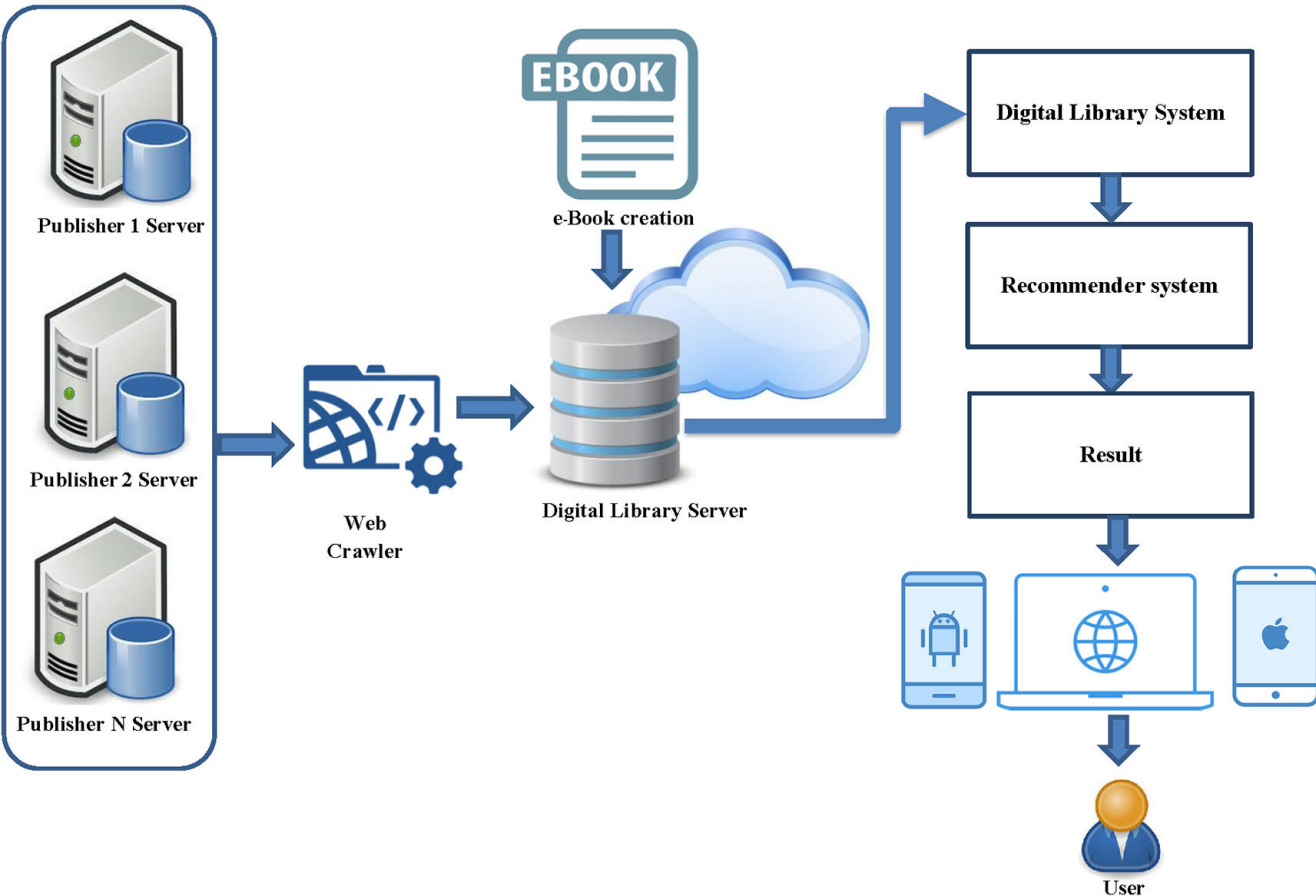
Framework of hybrid recommender system.

This part describes the framework of hybrid recommender system including API function for connect multiply publisher, architecture of the book recommendation system, hybrid recommender systems model. The concept of hybrid recommender system was shown in Figure 1. This is a functional overview of a hybrid recommender system for a digital library from multiple online publishers. The system collects data from various publishers by creating a retrieval API and gathers important metadata for indexing. The metadata of various e-books are stored in the database of the developed system, without storing the e-book file from the publisher to maintain the book’s copyright. Partnered publishers for this edition of the book collection include the Listing Agency, Arsom Silp Institute of the Arts, and The Secretariat of the House of Representatives, all of which are valuable books in Thailand. The next step is to develop a digital library system with a channel for accessing book information. The login will be in the form of a one-time login for users to access all book listings linked to the system. The final step is to develop a recommendation system in the form of a hybrid recommender system and present the recommendation results to the user.

### 3.1 Function for connect multiply publisher

The process of collect Mata data from other sources. The system will link the book information through the database of the service provider and crawl data to collect information on each book for a created index such as title, category details, URL, etc. Therefore, users can read the original E-book through the URL of the book provider directly to support copyright from each E-book database policy. This prototype had a wide variety of e-books from a different database of organizations. All organizations encouraged Thai people to have access to reading services research information free of charge by creating functions to connect E-book data. However, the function may be adjusted according to the connection characteristics of different database systems. Initially, the system pass parameters required by the service and return values for data use as the following API functions including:

• Login function: The Login function supports user login and user logout.
• Get books list function: This function retrieves a list of all books purchased by the agency, along with basic information such as the title, author, publisher, number of pages, and number of copies.
• Get Category function: This function retrieves a list of book categories that the agency purchases, along with the number of books in each category.
• Get books by category function: This function retrieves a list of books in a specific category, along with basic information such as the title, author, publisher, number of pages, and number of copies.
• Get book type function: This function retrieves a list of book types that the agency purchases, along with the number of books of each type.
• Get books by book type function: This function retrieves a list of books in a specific book type, along with basic information such as the title, author, publisher, number of pages, and number of copies.
• Get book detail function: This function retrieves detailed information about a specific book, such as the title, author name, publisher, ISBN, year of publication, number of pages, number of volumes, and description.
• Search books function: This function searches for books available in the system based on the search query, which can be by title, author, publisher, or description.
• Read book function: This function checks the number of books that can be opened for reading.
• Checkout function: This function checks the number of books that can be checked out for online borrowing.

Table 1 show algorithm provides a structured approach to creating an API that manages user authentication, retrieves book details, and facilitates book reading functionality in a digital library system with multiple publishers. The emphasis is on securing user sessions, validating access permissions, and integrating external publisher APIs for a comprehensive digital library service.

**Table 1.** Algorithm of API for connection Multi-Publisher.

Algorithm: Develop API for Book Reading in a Multi-Publisher Digital Library System	
**Input:** User credentials, Book ID **Output:** Access to the book content for reading	
**Step 1:** API Endpoint for User Authentication FUNCTION authenticateUser (username, password) VERIFY user credentials IF credentials are valid GENERATE a user session token RETURN session token ELSE RETURN authentication error END IF END FUNCTION **Step 2:** API Endpoint for Retrieving Book Details FUNCTION getBookDetails (bookId, sessionToken) VALIDATE sessionToken IF sessionToken is valid SEARCH for bookId in the local database IF bookId exists in the local database RETRIEVE book details from the local database RETURN book details ELSE CALL externalPublisherAPI (bookId) RETRIEVE book details from the publisher's API STORE retrieved book details in the local database RETURN book details END IF ELSE RETURN session validation error END IF END FUNCTION	**Step 3:** API Endpoint for Reading Book Content FUNCTION readBookContent (bookId, sessionToken) VALIDATE sessionToken IF sessionToken is valid RETRIEVE the book content URL or data for bookId CHECK copyright and access permissions IF access is granted PROVIDE access to the book content LOG the reading activity RETURN book content access ELSE RETURN access denied message END IF ELSE RETURN session validation error END IF END FUNCTION **Step 4:** External Publisher API Interaction FUNCTION externalPublisherAPI (bookId) SEND a request to the publisher's API with bookId RECEIVE book details from the publisher's API RETURN book details to the calling function END FUNCTION End

### 3.2 The architecture of the book recommendation system

**Figure 2.**
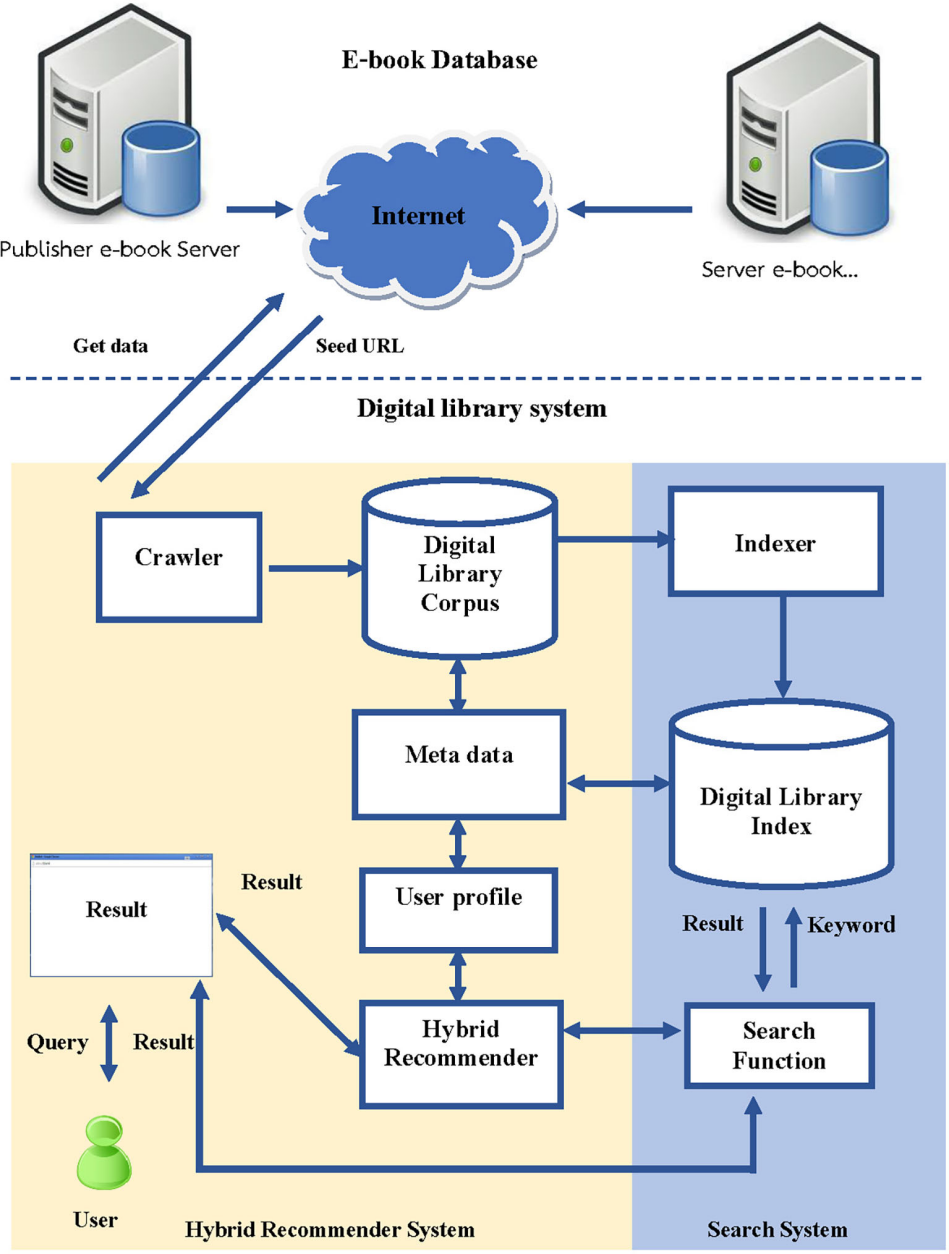
The architecture of the hybrid book recommendation system development of the online library system.

The architecture for developing the book recommendation system in the digital library consists of several steps, which are illustrated in Figure 2:

• *Crawler Data* is a detail within the session that connects multiple publishers. The research develops programs responsible for extracting data from online databases and storing it in a database. The system collects the following information: title, author, date, month, year of publication, and ISSN, which is useful for monitoring user interest and indexing each e-book.
• *Digital Library corpus* is a database used to store details of books that Crawler retrieves from an authorized database system and is an e-book database system developed by the library itself.
• *User Profile* is created by storing information about each user’s reading behaviour, such as books they have read, books they have selected for their shelf, and books they have rated or reviewed, and these data are then processed to find out which books and what categories the user likes or dislikes in order to bring information to be fed to the Recommender System to recommend other books that are similar in content or genre to the books the user has already read and enjoyed. The system can also suggest books based on the user’s reading history and preferences, such as authors or topics they have shown interest in.
• *Hybrid recommender system* Combines the recommendations from Content-Based Filtering and Collaborative Filtering to generate a final list of personalized book recommendations for the user. The details are described in the next Session.

User Profiles can be stored and collected in the form of implicit feedback, including which books users view details and place on their personal bookshelves. Creating user profiles is a process of building a model of user settings. Assuming that there are *n* users participating in the system, *m* is books have been read, *o* is books have been keep in user shelf, and *p* is books have been reviewed.

Let *U* be a set of all the users contained in the system; *U* = { *U*_1_, *U*_2_ …, *U _n_*}, *R* is a set of books read from digital library collection; *R* = { *r*_1_, *r*_2_ •••, *r _m_*}, *K* is a set of keep from digital library collection; *K* = { *k*_1_, *k*_2_ •••, *k _o_*}, *V* is a set of rating; *V* = { *v*_1_, *v*_2_ •••, *v _p_*}, *URKV _ijal_* is a set of user read books and keep book and rating book by user *U _i_; URKV _ijkl_* = { *urkv*_*ilal*_, *urkv*_*i*2 *al*_, …, *urkv _ijal_*} and Let *E* ( *u _i_, urkv _ijal_*) indicates a relationship among user *U _i_,* with read *UR _ij_.* Here is the definition of the user profile.:

Definition [User Profile]:

For *a* user *p _i_* where *i* = 1, .., *n*;

Let *U _i_*; be a user profile of user *u _i_.*

*U _i_*; = { *< u _i_, urkv _ijal_>/urkv _ijal_* ∈ *URKV^ u _i_* ∈ *U ^ E* ( *u _i_, urkv _ijal_*) = 1}

When *a *new user signs up for the digital library system, the recommender system may not be able to generate accurate recommendations since there haven’t been any interactions between the user and the books. Additionally, if the model hasn’t been updated since the user’s registration, the system may not recognize their existence and thus cannot make any predictions for them through CF. To resolve these problems, during the registration process, users are required to select one to three preferred categories. This information is used by a customized content-based filtering algorithm to provide personalized recommendations until the CF model can generate high-quality recommendations based on the user’s interactions.

### 3.3 Hybrid recommender systems model

A hybrid recommender system is a process that introduces e-books by analyzing data from users’ reading behavior. The system utilizes a combination of Collaborative Filtering (CF) and Content-Based Filtering (CB) to recommend e-books to users. This involves applying a weighted score to the recommendations generated by each of these methods. The process of hybrid recommending e-books to individual users is designed to suggest related e-books or e-books that users are expected to like. This is done by considering the User Profile that is collected from the user. The User Profile includes a set of user read books, the books that are kept in the shelf, and ratings given by the user to different books.

• Collaborative Filtering (CF) is used to identify users who have similar preferences and interests based on their reading behavior. This involves analyzing the behavior of similar users to identify e-books that the user might be interested in. The User Profile is used to identify similar users who share similar interests and preferences. This method is effective in generating recommendations for users who have similar reading habits. The maximum score of user similarity is one. Collaborative Filtering score is showed in equation 1.
$${\hat{r}}_{\mathrm{ib}}={\bar{r}}_{i}+\frac{\sum_{u_{j}\in U,j\neq i}\text{similarity}\left(U_{i},U_{j}\right).\left(r_{\mathrm{jb}}-{\bar{r}}_{j}\right)}{\sum_{u_{j}\in U,j\neq i}\left|\text{similarity}\left(U_{i},U_{j}\right)\right|}$$ (1)

${\hat{r}}_{\mathrm{ib}}$ is the predicted preference or rating for book *b* by user *i*

${\bar{r}}_{i}$ is the average rating or preference of user *i* based on their interactions

$r_{\mathrm{jb}}$ is the rating or interaction of user *j* with book *b*

${\bar{r}}_{j}$ is the average rating or preference of user *j*

$\text{similarity}\left(U_{i},U_{j}\right)$ is the similarity score between the profiles of users *i* and *j*
• Content-Based Filtering, on the other hand, recommends e-books based on the factors that the user has liked in the past. This involves analyzing factors such as the category of books, the publisher, and the year of publication. This method is useful for recommending e-books that match the user’s specific preferences. All of three factors are combined and maximum score is one. The detail of each score as follow:
  1) Category of books Score: The definition of $P_{\text{category}}$ as a set of book categories that the user likes, and *C* as the category of the book being considered, allows us to calculate the score for the book category. This can be utilized in a Content-Based Filtering recommendation system to suggest books that align with the user’s preferences, as shown in equation 2.
$$\text{Category of books Score}=\left\{ \begin{array}{ll} 1, & \text{if}c\in P_{\text{category}}\\ 0, & \text{Otherwise} \end{array}\right.$$ (2)
    This means that If the category of the book being considered ( *C*) is within the set of categories that the user likes ( $P_{\text{category}}$ ), the book will receive a score of 1, indicating high relevance to the user. If the book’s category is not within the user’s preferred category set, it will receive a score of 0, indicating low or no relevance to the user. Using category score in the recommendation system helps to accurately suggest books that match the user’s interests and preferences in specific book categories.
  2) Publisher score: The definition of $P_{\text{publisher}}$ as the set of publishers that the user prefers, and *P* as the publisher of the book under consideration, we can use this information to calculate the score for the publisher, which can be utilized in a Content-Based Filtering recommendation system to suggest books from publishers that the user likes, as shown in equation 3.
$$\text{Publisher score}=\left\{ \begin{array}{ll} 1, & \text{if}\;P\in P_{\text{publisher}}\\ 0, & \text{Otherwise}\; \end{array}\right.$$ (3)
    This means that If the publisher of the book under consideration ( *P*) is within the set of publishers that the user likes $P_{\text{publisher}}$ the book will receive a score of 1, indicating high relevance to the user. If the book’s publisher is not within the user’s preferred set of publishers, the book will receive a score of 0, indicating low or no relevance to the user. Moreover, using publisher score in the recommendation system helps to accurately suggest books from publishers that match the user’s preferences and past positive experiences, enhancing the personalized recommendation process.
  3) Year of publication score: Year of Publication Score emphasizes the book’s novelty. The scoring process evaluates by contrasting the publication year with the current year. Previously, there has been research that has integrated the year factor with other elements to facilitate book recommendations, ^37^ as shown in equation 4.
$$\text{Year of publication score}=\frac{n}{\arg_{n}\max\left(n\right)}$$ (4)
  4) Content-Based Filtering score: this score (CB *Score*) is calculated as the average from of the Category Score, and the Publisher Score and the Year of Publication Score. Each of these scores contributes to assessing the relevance of a book based on its publication date, the category it belongs to, and the publisher. By adding these scores together and dividing by three, the CB score provides a comprehensive metric that reflects the book’s overall alignment with a user’s preferences in terms of recency book, genre, and the credibility or popularity of the publisher as shown in equation 5.
$$\mathrm{CB}-\text{Score}=\frac{\left(\text{Category Score}+\text{Publisher Score}+\text{Year of publication Score}\right)\;}{3}$$ (5)

To generate a final list of personalized e-book recommendations for the user, the recommendations generated by both Content-Based Filtering and Collaborative Filtering are combined. The system uses a weighting scheme to determine the relevance of each recommendation, based on factors such as the user’s past behavior, the popularity of the e-book, and other relevant factors. This results in a list of e-books that are tailored to the user’s interests and preferences, increasing the likelihood that the user will find e-books that they enjoy reading. Here is a formula for a hybrid recommender system that merges collaborative filtering and content-based filtering techniques:

$$\text{Hybrid Score}=\left(1-\alpha \right)\times \;\text{CF-Score}+\alpha \times \;\text{CB-Score}$$ (6)

Such as:

$$\text{Hybrid Score}50:50={\left(0.5\right)}^{}\times \;\text{CF-Score}+0.5\times \;\text{CB-Score}$$

$$\text{Hybrid Score}20:80=\left(0.2\right)\times \;\text{CF-Score}+0.8\times \;\text{CB-Score}$$

$$\text{Hybrid Score}80:20={\left(0.8\right)}^{}\times \;\text{CF-Score}+0.2\times \;\text{CB-Score}$$

where:

*CF-Score* = similarity between the target user and other users who have similar preferences

*CB-Score* = relevance score of recommended items based on their content

*α* = a weighting factor that determines the relative importance of the two scores

**Figure 3.**
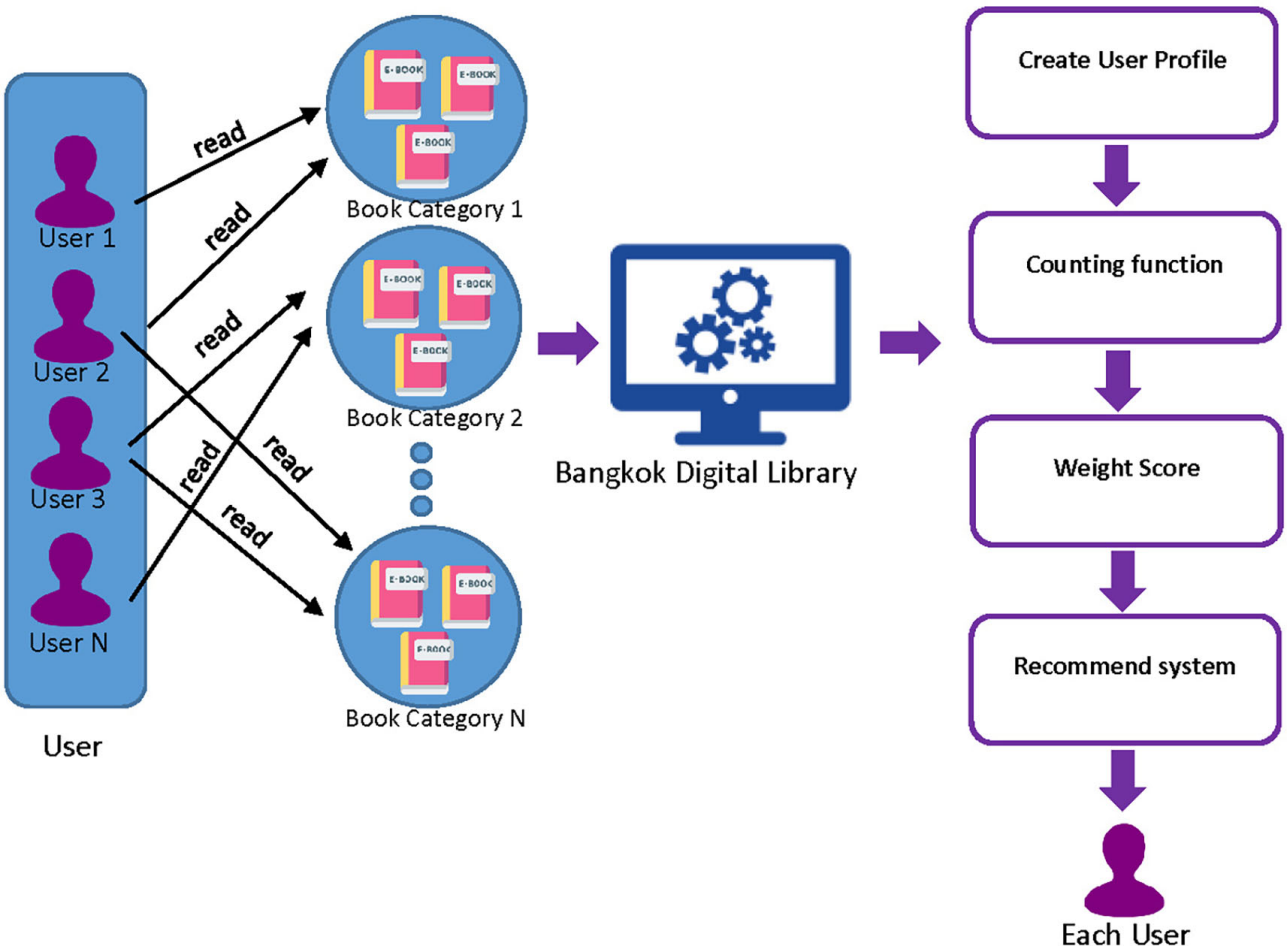
The process of hybrid recommender systems model.

## 4. Experimental approach

The environment in which the experiment is conducted is split into three distinct parts. The first section describes the data set, the second describes the evaluation metric, and the last section describes the experimental results.

### 4.1 The digital library corpus

The collection of E-books comprises 2,715 items, while the number of members registered is 370 members from Library System for Learning in 2022. The digital library dataset includes the following information for each item: book ID, title, description, keywords, book categories, keywords, and book details, category of books, the publisher, and the year of publication, and either an e-book file in the owner’s system or a URL for accessing the full e-book in the case of books from partners.

### 4.2 Evaluation metric

To address the proposed experiment, this research carried out a study by inviting general users to participate in an evaluation. This aligns with the research on a hybrid approach to knowledge recommender services as documented in study. ^38^ In the experimental setup, the research participants were assigned the task of exploring books from the digital library. The seventy five subjects, specifically members of the general public who were interested in reading digital books and were proficient in using applications were invited and participated in the evaluation. Each participant was given six different search queries, and all queries were tested using different ranking approaches. The search engines presented the top 15 documents according to their relevance, with *i* representing the ranking number { *i* = 1, 2, 3, …, 15}. The participants were then asked to rate the relevancy of the search results using a five-point scale: Score 0 indicating “not relevant at all,” Score 1 indicating “probably not relevant,” Score 2 indicating “less relevant,” Score 3 indicating “probably relevant,” and Score 4 indicating “extremely relevant.” This paper utilized the Normalized Discounted Cumulative Gain (NDCG) metric to measure the performance of every search engine. ^39^ This measurement is specifically designed for evaluating web search performance. The NDCG was calculated using the equation (7).

$${\text{NDCG}}_{q}=M_{q}\sum_{j=1}^{k}\frac{\left(2^{r\left(j\right)}-1\right)}{\log\left(1+j\right)}$$ (7)

The parameter *k* represents the truncation or threshold level, while the integer *r*( *j*) denotes the relevancy score given by the research participant. The normalization constant *M _q_* is calculated to ensure that the ideal ordering would achieve an NDCG score of 1. The NDCG metric emphasizes relevant documents that appear among the top search results while penalizing irrelevant documents by reducing their impact on the NDCG score.

### 4.3 Experimental results

**Figure 4.**
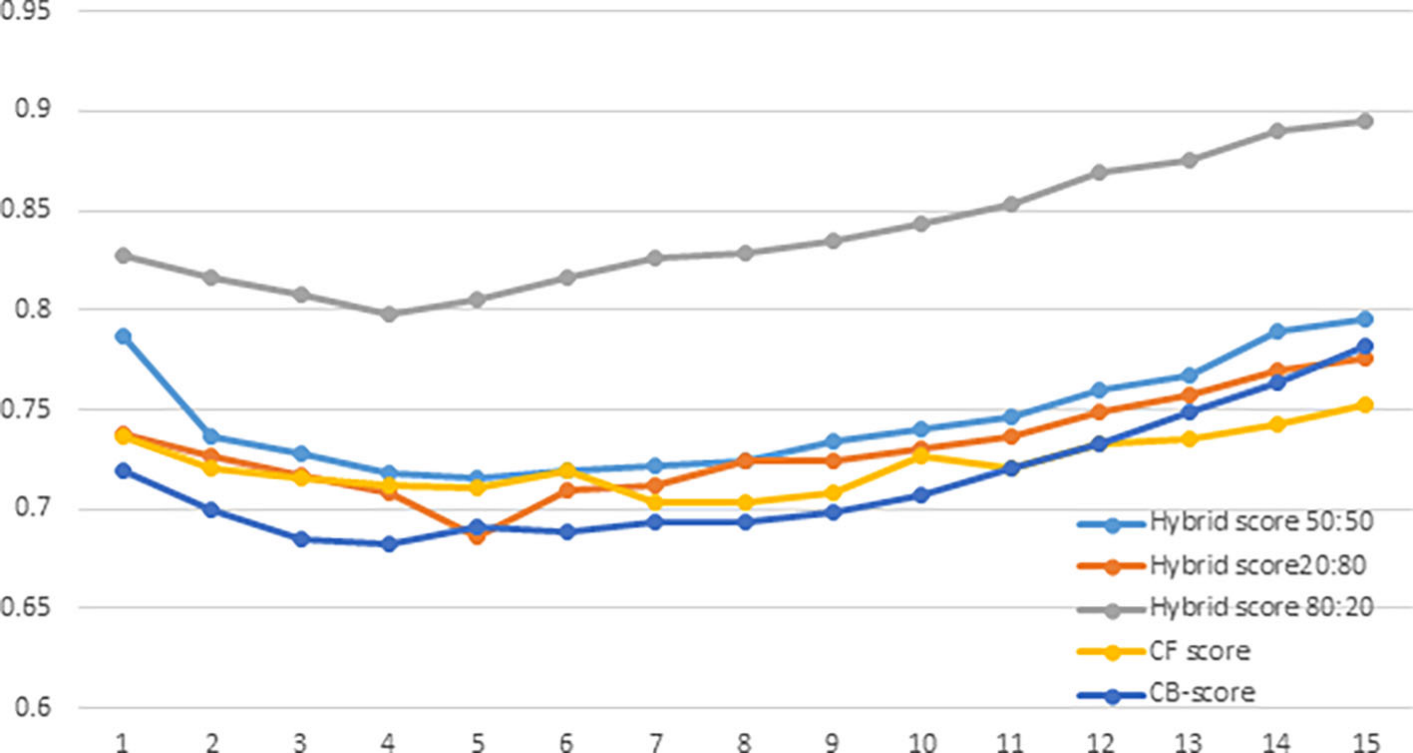
Comparison of the average NDCG score.

User evaluation refers to the process of collecting feedback from users on the performance of a recommender system. NDCG average score is a metric used to evaluate the performance of the system, calculated by taking the average of the NDCG scores for all users in the dataset. A comparison of NDCG of *Hybrid Score50:50*, *Hybrid Score20:80, Hybrid Score80:20, CF-score* and *CB-score* are shown in Figure 4 *. CF-score* and *CB-score* are standalone recommendation algorithms that use either *CF* or *CB* exclusively. The study compares the average NDCG scores of five distinct recommender approaches. The graph has the x-axis representing the top 15 ranks of the search results and the y-axis displaying the NDCG score. Based on the graph, it appears that the *Hybrid Score80:20* method has the highest NDCG average score among the five different recommender approaches being compared. This suggests that the *Hybrid Score80:20* algorithm is the most effective at recommending relevant items to users.

This research applied One Way ANOVA on NDCG at top fifteen ranks ( *K* = 1, 1-2, 1-3,…, 1-15) respectively to test whether there is a difference among the mean NDCG from three different recommender system model approaches. The researcher set up the hypothesis that the is no statistically significant difference between the *CF-score* and *CB-score*. The result indicates that the means of NDCG for the tree approaches to recommender system models were not equal with a significance level of α = 0.05. In simpler terms, there was a statistically significant difference in the search results. From Table 2 statistically significant differences were observed in the search results between the *CB-score* and *CF-score*, and between the *CB-score* and *HybridScore 80:20*.

**Table 2.** Result of multiple comparisons.

Rank (K)	Indexing		Mean Difference (I-J)	Std. Error	Sig. (2-tailed) (I)
1-15	*CB-score*	*CF-score*	-0.38	0.287	0.014
		*Hybrid Score*80:20	-0.25	0.081	0.003
1-15	*Hybrid Score*80:20	*CF-score*	0.47	0.052	0.276
		*CB-score*	0.25	0.081	0.003

## 5. Conclusion

The main focus of this research paper is the utilization of a heuristic recommender system that utilizes a Hybrid model. Seventy five participants were involved in the study from general public, and each participant generated six queries to investigate the e-books obtained through the recommender system. The top 15 documents for each search engine were displayed for relevance, and the participants rated the search results on a five-point scale based on relevancy. The results of the study indicate that the Hybrid model outperforms other models with a higher NDCG score, which suggests that the *Hybrid Score80:20* performs better than other recommender models. Additionally, a One Way ANOVA was used to further analyze the mean difference results of *CF-score* and *CB-score.* The statistical testing results indicate that the mean NDCG scores differ among the Hybrid model, *CF-score*, and CB-score at k = 1-15. However, the mean NDCG scores do not differ between the Hybrid model and CF-filtering. The study suggests that further experimentation should be conducted to explore different Hybrid models. The process of aggregating data from multiple online publishers is a formal procedure that necessitates close collaboration and coordination with various agencies and publishing entities to secure comprehensive and accurate data. Specific challenges such as disparities in data formats, access rights, and data privacy protection must be effectively managed during the system’s development. These details are crucial for the system to leverage diverse data sources to generate valuable and relevant recommendations for users. The paper has some limitations such as the sample size of 75 participants, which may not be representative of the wider population, and may limit the generalizability of the study findings. Additionally, the participants in the study may have had different levels of familiarity with the e-books, which could have influenced their ratings of relevancy. Moreover, the study highlights the importance of using a hybrid model to improve the effectiveness of recommender systems. Future research should delve into the capabilities of deep learning techniques to augment the personalization aspect of the hybrid model.

## Data Availability

This research cannot provide the underlying data because it involves copyrighted data from multiple publishers, and all publishers have agreements that prohibit developers from disseminating book information and user experimentation data under the principles of the Personal Data Protection Act (PDPA). The data set was sourced from the Bangkok Digital Library System at https://www.bangkoklibrary.go.th/digital/. To access the dataset, please contact us via email at addigitallibrarybkk@gmail.com. Figshare: Evaluation form for Subject Test.pdf. https://doi.org/10.6084/m9.figshare.22308823.v1. ^40^ This project contains the following extended data:
- Evaluation form for Subject Test.pdf Data are available under the terms of the Creative Commons Attribution 4.0 International license (CC-BY 4.0).
