# Supplementary material for: Hybrid recommender system model for digital library from multiple online publishers
Source: F1000Res. 2024 Nov 18;12:1140. Originally published 2023 Sep 12. [Version 3] doi: 10.12688/f1000research.133013.3 (PMC11739703; doi:10.12688/f1000research.133013.3)
Supplement: Peer review history

# Reviewer report: Reviewer response for version 3

**DOI:** 10.5256/f1000research.174529.r347047

**Published:** 2025-01-16

**Authors:** Monika Verma

**Affiliations:** 1 Bhilai Institute of Technology, Bhilai, Chhattisgarh, India

Major:

 1) Data Source should be provided for better understanding of methodology

 2) The methodology section lacks sufficient detail on how the sample was selected, which raises concerns about the generalizability of the results. Methodology need to be discussed in better detail.

 3) Increase Sample size for performance evaluation

 4) use at least one more performance metric to evaluate

 Minor:

 1) Cite few good papers , for eg add below references (Refer 1 and 2) and cite them properly in section 2

Is the work clearly and accurately presented and does it cite the current literature?

Partly

If applicable, is the statistical analysis and its interpretation appropriate?

I cannot comment. A qualified statistician is required.

Are all the source data underlying the results available to ensure full reproducibility?

Yes

Is the study design appropriate and is the work technically sound?

Yes

Are the conclusions drawn adequately supported by the results?

Partly

Are sufficient details of methods and analysis provided to allow replication by others?

Yes

Reviewer Expertise:

Recommender System, machine learning, Deep learning, Text Mining, Academic recommender system

I confirm that I have read this submission and believe that I have an appropriate level of expertise to confirm that it is of an acceptable scientific standard, however I have significant reservations, as outlined above.

## References

[1] : An automatic college library book recommendation system using optimized Hidden Markov based weighted fuzzy ranking model. *Engineering Applications of Artificial Intelligence* .2024;130: 10.1016/j.engappai.2023.107664 10.1016/j.engappai.2023.107664

[2] : An Enhanced Item-Based Collaborative Filtering Approach for Book Recommender System Design. *ECS Transactions* .2022;107(1) : 10.1149/10701.15439ecst 15439-15449 10.1149/10701.15439ecst

# Reviewer report: Reviewer response for version 3

**DOI:** 10.5256/f1000research.174529.r341399

**Published:** 2024-11-20

**Authors:** Asefeh Asemi

**Affiliations:** 1 Corvinus University of Budapest, Budapest, Hungary

Author revisions are acceptable.

Is the work clearly and accurately presented and does it cite the current literature?

Partly

If applicable, is the statistical analysis and its interpretation appropriate?

Yes

Are all the source data underlying the results available to ensure full reproducibility?

Partly

Is the study design appropriate and is the work technically sound?

Partly

Are the conclusions drawn adequately supported by the results?

Partly

Are sufficient details of methods and analysis provided to allow replication by others?

Partly

Reviewer Expertise:

Library and Information Science, Recommender Systems

I confirm that I have read this submission and believe that I have an appropriate level of expertise to confirm that it is of an acceptable scientific standard.

# Reviewer report: Reviewer response for version 2

**DOI:** 10.5256/f1000research.164054.r263085

**Published:** 2024-07-23

**Authors:** Asefeh Asemi

**Affiliations:** 1 Corvinus University of Budapest, Budapest, Hungary

As a reviewer, there are several suggestions to improve the paper. Firstly, the methodology section could be condensed to provide a succinct overview of the research approach, focusing on essential details such as data collection, system development, and experimental design. This would help maintain a better balance between sections and improve readability. Secondly, the evaluation of the hybrid recommender system could be strengthened by increasing the sample size of participants and incorporating additional evaluation metrics to provide a more comprehensive assessment of system performance. Furthermore, discussing the limitations of the study in more detail and exploring their implications for the findings would enhance the paper's credibility. Additionally, providing clearer statements regarding the contribution of the proposed hybrid recommender system compared to existing literature or systems would help clarify the paper's significance.

Is the work clearly and accurately presented and does it cite the current literature?

Partly

If applicable, is the statistical analysis and its interpretation appropriate?

Yes

Are all the source data underlying the results available to ensure full reproducibility?

Partly

Is the study design appropriate and is the work technically sound?

Partly

Are the conclusions drawn adequately supported by the results?

Partly

Are sufficient details of methods and analysis provided to allow replication by others?

Partly

Reviewer Expertise:

Library and Information Science, Recommender Systems

I confirm that I have read this submission and believe that I have an appropriate level of expertise to state that I do not consider it to be of an acceptable scientific standard, for reasons outlined above.

# Response

**Published:** 2024-11-06

**Authors:** pijitra jomsri

**Affiliations:** Information Technology, Suan Sunandha Rajabhat University, Bangkok, Bangkok, Thailand

Thank you very much for the suggestions. I have implemented the recommended revisions as follows:

1. The **API functions** section has been condensed, while other sections have been expanded, as recommended by the committee, to provide clearer details on the model itself.
2. **Data collection** was expanded from 30 to 150 participants to enhance the credibility of the research findings. **Additional evaluation metrics**, specifically **precision**, were incorporated for further assessment, as detailed in Section 4. I have also elaborated on the **limitations** in Section 5, Note that the user base for eBook services in these libraries remains limited in Thailand, which led to an extended data collection period.
3. I have added content to the **conclusion** section and provided a more comprehensive comparison with existing research in Section 5 to clarify how this study aligns with previous findings.

# Reviewer report: Reviewer response for version 1

**DOI:** 10.5256/f1000research.145977.r222585

**Published:** 2023-11-30

**Authors:** Muhammad Yousuf Ali

**Affiliations:** 1 Aga Khan University, Karachi, Karachi, Sindh, Pakistan

I appreciated the Authors research work about the recommender system. However, following the following points are recommend to improve the readership and audience of this article. 

 1. Research objective is the one of the key point to carry out/conduct any research but authors did not mentioned research objective. Recommend add this research objective.

 2. In this research paper researcher(s) did not mention what the research questions are and they try to explore the answer to the Research questions or hypothesis they are trying to test. 

 2.The Methodology section is missing the basic concept of the research. The authors try to carry out Quasi Experimental designed research, but did not express this methodology and not include any citations. 

 3. N=30, as the authors mentioned in section 5.0 line 1, did not define the population/sample characteristics. Define the sample like students, researchers, general public, children or women take part in this study. 

 4. "Experimental Results" Section 4.3, the authors applied a One way ANOVA test. I will recommend to draw a non-directional hypothesis that: "Is there any significant relationship between the Content filtering (CF) score and Content base (CB) score", and you validate your hypothesis accordingly.

Is the work clearly and accurately presented and does it cite the current literature?

Yes

If applicable, is the statistical analysis and its interpretation appropriate?

Partly

Are all the source data underlying the results available to ensure full reproducibility?

Yes

Is the study design appropriate and is the work technically sound?

Partly

Are the conclusions drawn adequately supported by the results?

Partly

Are sufficient details of methods and analysis provided to allow replication by others?

Partly

Reviewer Expertise:

NA

I confirm that I have read this submission and believe that I have an appropriate level of expertise to confirm that it is of an acceptable scientific standard, however I have significant reservations, as outlined above.

# Response

**Published:** 2024-06-20

**Authors:** pijitra jomsri

**Affiliations:** Information Technology, Suan Sunandha Rajabhat University, Bangkok, Bangkok, Thailand

I am deeply grateful for your valuable guidance on improving my paper.  I am pleased to inform you that ***I have revised the paper (Version 2)*** according to all your recommendations. Here are the details of the revisions as follow:

 1. **Objective of Paper**: Added the objective, "To develop a digital library model by studying techniques for combining multiple e-book database systems," which now appears in Section 1. Introduction.

**2.Research Questions:** Added the research question, "The researcher believes that a hybrid recommender system model can enhance the efficiency of book recommendations in the digital library system," also in Section 1. Introduction.

**3. Methodology :** Expressed detailed methodology and added citations in Section 4.2 Evaluation Metric.

**4. Sample Characteristics:** Defined the characteristics of the sample and increased the sample size from 30 to 75 participants, as specified in Section 4.2 Evaluation Metric.

**5. Hypothesis:** In Section 4.3, I have formulated the hypothesis as recommended by the reviewer.

# Reviewer report: Reviewer response for version 1

**DOI:** 10.5256/f1000research.145977.r216613

**Published:** 2023-11-10

**Authors:** Asefeh Asemi

**Affiliations:** 1 Corvinus University of Budapest, Budapest, Hungary

Clarity and Accuracy: The text is well-organized and provides a clear structure for the research paper. The abstract, introduction, methodology, and results sections are clearly defined. 

 Study Design and Technical Soundness: The study design seems appropriate for the research question of developing a hybrid recommender system for a digital library. The technical components, such as the API connection and hybrid recommender system model, are well-described.

 Details of Methods and Analysis: The methods section provides an overview of the digital library corpus, evaluation metric (NDCG), and experimental results. However, specific details about the algorithms used in Collaborative Filtering (CF) and Content-Based Filtering (CB) are not provided. More information on these aspects would enhance the clarity.

 Statistical Analysis: The use of the Normalized Discounted Cumulative Gain (NDCG) as an evaluation metric is appropriate for assessing the performance of the recommender system. The statistical analysis, particularly the One Way ANOVA, adds a quantitative aspect to the evaluation.

 Source Data Availability: The authors mention that the underlying data cannot be provided due to copyright restrictions from multiple publishers. However, they offer a link to the Bangkok Digital Library System for accessing the dataset.

 Support for Conclusions: The conclusion suggests that the Hybrid Score 80:20 method outperforms other models. The provided graph supports this claim.

 Constructive Feedback: To enhance clarity, provide more details about the algorithms used in Collaborative Filtering (CF) and Content-Based Filtering (CB). Consider expanding on the limitations of the study, especially regarding the small sample size of 30 participants and potential biases in their familiarity with e-books. Encourage the authors to provide more context on the specific challenges faced in connecting data from multiple online publishers and how these challenges were addressed in the development of the hybrid recommender system.

 The work appears promising, but additional details, especially regarding the specific algorithms used, and further context on challenges faced during implementation would contribute to a more comprehensive understanding of the research.

Is the work clearly and accurately presented and does it cite the current literature?

Partly

If applicable, is the statistical analysis and its interpretation appropriate?

Yes

Are all the source data underlying the results available to ensure full reproducibility?

Partly

Is the study design appropriate and is the work technically sound?

Partly

Are the conclusions drawn adequately supported by the results?

Partly

Are sufficient details of methods and analysis provided to allow replication by others?

Partly

Reviewer Expertise:

Library and Information Science, Recommender Systems

I confirm that I have read this submission and believe that I have an appropriate level of expertise to confirm that it is of an acceptable scientific standard, however I have significant reservations, as outlined above.

# Response

**Published:** 2024-06-20

**Authors:** pijitra jomsri

**Affiliations:** Information Technology, Suan Sunandha Rajabhat University, Bangkok, Bangkok, Thailand

Thank you for your valuable guidance on improving my paper. I am pleased to inform you that I have revised the paper (Version 2) according to your recommendations. The following improvements have been made such as:

 1. Increased Sample Size: The sample size has been increased to 75 participants to expand the dataset and provide additional comparative results. The experimental findings, as shown in Table 2, indicate that the Hybrid Score 80:20 still performs better.

 2. Enhanced Algorithm Details: Additional details about the algorithms have been provided in Section 3.1 (Function for Connecting Multiple Publishers) and Section 3.3 (Hybrid Recommender Systems Model) to clarify the methodology.

 3. Challenges and Solutions: Additional information regarding the challenges faced in connecting data from multiple online publishers has been included in the Conclusion.
